# Reactions to Unsolicited Violent, and Sexual, Explicit Media Content Shared over Social Media: Gender Differences and Links with Prior Exposure

**DOI:** 10.3390/ijerph17124296

**Published:** 2020-06-16

**Authors:** Laura Louise Nicklin, Emma Swain, Joanne Lloyd

**Affiliations:** 1 Cyberpsychology Research Group, Department of Psychology, Faculty of Education, Health and Wellbeing, University of Wolverhampton, Wolverhampton WV1 1LY, UK; Laura.nicklin@wlv.ac.uk; 2 Department of Psychology, University of Wolverhampton, Wolverhampton WV1 1LY, UK; e.swain@wlv.ac.uk

**Keywords:** explicit content, social media, sexual, violent, unsolicited, cyberpsychology

## Abstract

While there has been extensive research into consumption of “traditional” forms of explicit sexual and violent media (within pornography, videogames and movies), the informal exchange and viewing of explicit real-world violent and sexual content via social media is an under-investigated and potentially problematic behaviour. The current study used an online survey (*n* = 225: 169f, 55m, 1x, mean age 30.61 (SD 12.03)) to explore self-reported reactions to unsolicited explicit violent and sexual content that participants had received from friends or contacts. In line with our predictions based on previous studies of fictional explicit content, we found effects of both gender and prior exposure on these reactions. Specifically, females rated both sexual and violent explicit content as significantly less funny and exciting and more disturbing than males did. Amongst males, those with high previous exposure rated violent content as more exciting than those with lower or no prior experience. Regardless of gender, participants with higher exposure to sexual content rated it as funnier than those with mild or no exposure, and those with higher exposure to violent content rated it as more amusing and more exciting. However, contrary to what desensitization theories would predict, prior exposure did not attenuate how disturbing explicit content (of either a sexual or a violent nature) was rated. Multiple avenues for further investigation emerged from this preliminary cross-sectional study, and we suggest priorities for further qualitative or longitudinal work on this novel topic.

## 1. Introduction

While there has been extensive research into the potential impacts of viewing fictional violent media in videogames and motion pictures [1], the informal exchange and viewing of explicit real-world violent footage is heavily under-researched, yet potentially highly problematic [2]. Viewing real-world footage of shootings or bombings on news media can initiate a “cycle of distress” and worry [3], but we know very little of the impacts of viewing explicitly violent real-world footage in an entirely uncensored way via online social networks. Anecdotal reports and media stories indicate that exchange and consumption of explicit violent imagery and video footage may be a normalised, peer-accepted cultural practice that some individuals enjoy as “entertainment”, but that others are passively exposed to, via receipt of unsolicited content from peers. Given that social media communication is highly pervasive [4], with image-based social media use, in particular, driven by fundamental human needs such as desire to fit in or belong [5], the infiltration of problematic, potentially disturbing content into these platforms, particularly when generated by peers, is a particular concern.

Exchange of explicit real-world sexual image-based content is also an important research priority [6], with the sharing of “dick pics”, for example, becoming so prevalent as to have been deemed an “emerging cultural practice” [7]. The context in which such content is shared influences whether it is experienced in a positive or negative way [8], with consent being of particular importance (i.e., whether sexual images are solicited or unsolicited; [9]). In addition to sexual motivations for sending such content, there are a variety of other commonly sought reactions, including shock, humour or disgust [9], which might motivate sharing amongst peers, alongside violent explicit content, under the auspices of “entertainment”. Research into online exposure to unsolicited pornographic material has often focused on content encountered through “pop up” advertisements or spam emails (e.g., [10]), whereas the current study sought to learn more about reactions to unsolicited sexual, alongside unsolicited violent, content shared amongst friends over social media.

Both individual and environmental factors have potential to influence reactions to such content. In particular, there is some evidence of gender differences in attitudes towards both sexual and violent content. Emmers-Sommer and colleagues conclude, based on self-report ratings, that it is “quite clear that men have a greater interest and desire to view violent and sexually violent film than women do” [11] (p. 313). Amount of prior exposure to explicit content is also thought to influence reactions to it, according to theories of desensitization via conditioning towards both violent media [12,13] and pornographic material [14,15].

The current study sought to explore, via online survey, how people react to the receipt of explicit violent and sexual content over social media, and to determine whether gender and prior exposure influence these reactions, as might be expected if unsolicited, real-world content induces similar responses to content purposely consumed in videogames, movies or pornography. We tentatively predicted that males, and/or those with more experience of receiving explicit content, would rate such content as less disturbing than females and/or those with less experience, and potentially as more amusing and exciting.

## 2. Materials and Methods

A convenience sample of active social media users aged 18 and above was recruited via an online (Qualtrics) survey, distributed across the social networks of the researchers. After reading an information sheet and providing informed consent online, respondents reported their age and gender, and indicated which social media platforms they currently use (see Table 1).

Participants were then given a definition of sexually explicit content and asked about their previous exposure to it; specifically, they were told:

*“The following questions will be referring to sexually explicit unwanted content. This could be any media such a pictures and videos containing sexual and/or pornographic details. Sexually explicit material (video, photography, creative writing) presents sexual content without deliberately obscuring or censoring it. The term “sexually explicit” is often used as euphemism for pornography. It includes real sex acts, sexual intercourse and uncovered genitalia. Every so often, friends or contacts will send sexually explicit pictures or videos without being asked. Have you ever personally been sent sexually explicit content, that you did not ask to see?”*

The response options were “never/not sure”; “yes, once or twice”; and “yes, multiple times”.

They were then asked to rate their agreement with three possible reactions to receiving such content: “I found it funny”; “I found it exciting”; and “I found it disturbing”. They were asked to rate based on how they thought they would feel if they had never actually received such content. Responses were on a seven-point Likert scale, with options “strongly disagree”; “disagree”; “somewhat disagree”; “neither agree nor disagree”; “somewhat agree”; “agree”; and “strongly agree”.

The same questions were repeated with reference to explicit unwanted violent content, which participants were told could be any media, such as pictures and videos containing violent details:

“The intentional use of physical force or power, threatened or actual, against oneself, another person, or against a group or community, that either results in or has a high likelihood of resulting in injury, death, psychological harm, maldevelopment, or deprivation.”

Upon completion of the survey, participants were provided with a debrief page containing contact details for mind.org, the University counselling service (for student participants), and the researchers’ contact details.

Ethical approval was granted by the University of Wolverhampton Psychology Ethics Committee.

## 3. Results

### 3.1. Sample

Of those who completed the survey, 169 were females (75.1%), 55 males (24.4%), and 1 “prefer not to say” (0.4%). The age of participants ranged from 19–36 years, mean 30.61 (SD 12.03). Overall, 54.6% (*n* = 123) were students. The percentage of respondents using each platform is summarised in Table 1; almost all used Facebook and WhatsApp.

### 3.2. Frequency of Receiving Explicit Content

**Figure 1:**
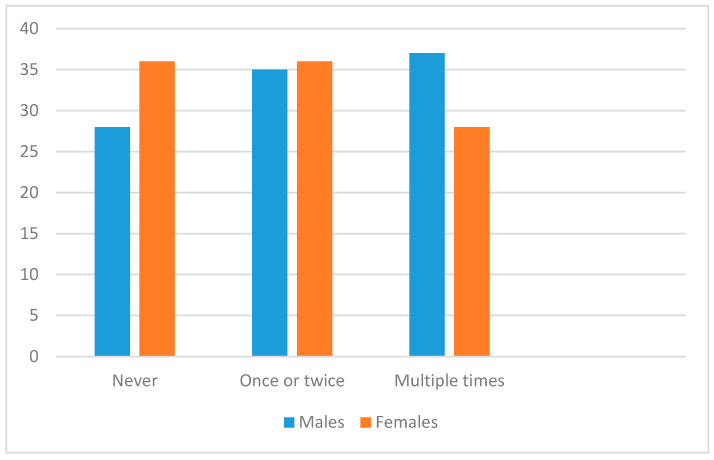
Frequency of prior exposure to unsolicited sexual content by males and females.

**Figure 2:**
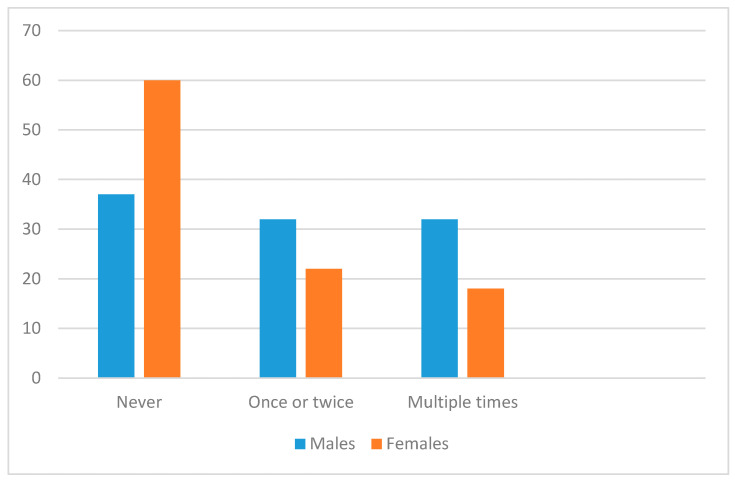
Frequency of prior exposure to unsolicited violent content by males and females.

As shown in Figure 1, over 2/3 of respondents had received explicit sexual content, with no major differences between males and females in each category (a chi-square test was non-significant, *p* > 0.05). Fewer respondents overall had received explicit violent content (see Figure 2), and distribution of females across categories here was significantly different from males (chi-square df 2 = 8.68, *p* = 0.013); over 60% of males had received violent content, compared with just 40% of females.

### 3.3. Reactions to Unsolicited Sexual Content

A series of three two-way, between-subjects ANOVAs were carried out to explore the effect of gender and prior exposure to unsolicited sexual content on participants’ ratings of how funny, exciting, and disturbing they deem such content to be.

There was a main effect of gender on all ratings of sexual content, with females rating it as significantly less funny (F(1213) = 19.67, *p* < 0.0005, η^2^ = 0.077), less exciting (F(1212) = 43.96, *p* < 0.0005, η^2^ = 0.167), and more disturbing (F(1211) = 24.86, *p* < 0.0005, η^2^ = 0.105) than males. Mean ratings by males and females are summarised in Table 2.

There was no main effect of frequency of receipt of sexual content on ratings of how exciting or disturbing such content was, and no interactions between gender and frequency of exposure (all *p*-values >0.05). There was a main effect of frequency of receipt of sexual content on ratings of how funny such content was found to be (F(2213) = 7.96, *p* < 0.0005, η^2^ = 0.062). Post-hoc t-tests revealed that those who had never received unsolicited sexual content rated it as significantly less funny (with a mean rating of 1.66, SD 1.98) than those who had received it once or twice (with a mean rating of 2.46, SD 2.02) (t (150) = −2.46, *p* = 0.015, d = 0.40), and less funny than those who had received it multiple times (who gave a mean rating of 3.13 (SD 1.96) (t (138) = −4.43, *p* < 0.0005, d = 0.75). The difference between occasional and multiple-time recipients was also significant (t (144) = −2.05, *p* = 0.042, d = 0.34).

### 3.4. Reactions to Unsolicited Violent Content

A further three two-way, between-subjects ANOVAs were carried out to explore the effect of gender and prior exposure to violent content on participants’ ratings of how funny, exciting, and disturbing they deem such content to be.

For ratings of how funny violent content was judged to be, there was no interaction between gender and prior exposure (*p* > 0.05), but there was a significant main effect of gender (F(1207) = 9.17, *p* = 0.003, η^2^ = 0.037), with females rating it as less funny than males, and a significant main effect of prior exposure (F(2207) = 12.20, *p* < 0.00005, η^2^ = 0.098). Post-hoc t-tests revealed that those who had never received violent content rated it as significantly less funny (with a mean rating of 0.43, SD 0.98) than those who had received it once or twice (with a mean rating of 1.25, SD 1.51) (Welch’s t (73.4) = −3.59, *p* = 0.001, d = 0.64), and significantly less funny than those who had received it multiple times (and who gave a mean rating of 1.68 (SD 1.94) (Welch’s t (56.10) = −4.20, *p* < 0.0005, d = 0.81). There was not a significant difference between those who received violent content a few times and those who received it many times (*p* < 0.05).

**Figure 3:**
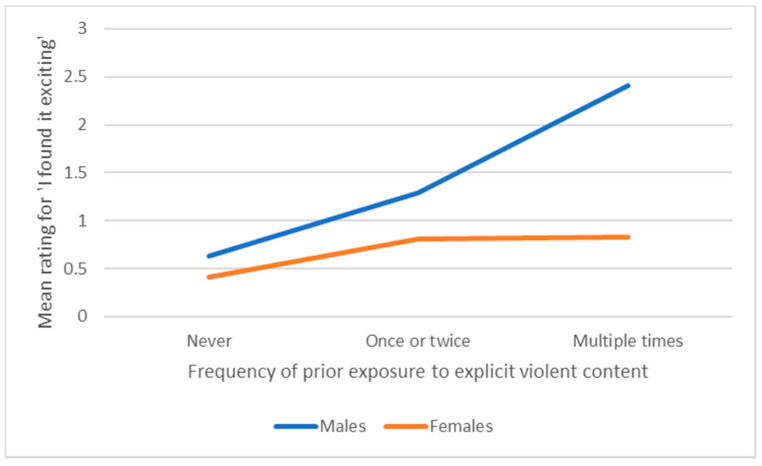
Interaction between gender and prior exposure on ratings of how exciting unsolicited violent content was perceived to be.

For ratings of how exciting violent content was, there was a significant main effect of gender, with females rating it less exciting than males (F(1207) = 14.83, *p* < 0.0005, η^2^ = 0.059); a main effect of prior exposure (F(2207) = 10.78, *p* < 0.0005, η^2^ = 0.086); and an interaction between gender and prior exposure (F(2207) = 4.32, *p* = 0.014, η^2^ = 0.035), as illustrated in Figure 3. Post-hoc one-way ANOVAs revealed that amongst females there was not a significant effect of frequency of prior exposure on ratings of how exciting violent content was (F(2157) = 2.58, *p* = 0.079 η^2^ = 0.032), whereas for males there was (F(250) = 6.66, *p* = 0.003 η^2^ = 0.210). T-tests revealed that although there was a tendency for males with occasional exposure to rate violent content as more exciting than those with no exposure (t (34)= −1.88, *p* = 0.069, d = 0.62), and less exciting than those with high exposure (Welch’s t (23.53) = −1.97, *p* = 0.061, d = 0.68), the only difference to reach significance was between those males who had never received violent content (rating it a mean of 0.63, SD 1.07) and those who had received it multiple times (who rated it a mean of 2.41, SD 2.09) (Welch’s t (23.17) = −3.16, *p* = 0.004, d = 1.07).

Post-hoc t-tests comparing ratings of excitement between those with different levels of exposure to violent content revealed that those who had never received violent content rated it as significantly less exciting (Mean 0.45, SD 0.89) than those who had occasionally received it (mean 0.96, SD 1.36) (Welch’s t (73.49) = −2.50, *p* = 0.015, d = 0.44) and those who had frequently received it (whose mean rating was 1.40, SD 1.79) (Welch’s t (55.63) = −3.48, *p* = 0.001, d = 0.67). The occasional and frequent recipients’ ratings did not differ significantly (Welch’s t (85.31) = −1.38, *p* = 0.172, d = 0.28).

For ratings of how disturbing violent content was perceived to be, there was a significant main effect of gender, with males rating it less disturbing than females (F(1207) = 8.19, *p* = 0.005, η^2^ = 0.037), but no main effect of prior exposure (F(2207) = 2.61, *p* = 0.076, η^2^ = 0.024) and no significant interaction between gender and prior exposure (F(2207) = 0.320, *p* = 0.726, η^2^ = 0.003).

## 4. Discussion

In this online survey study, we explored how often social media users had received unsolicited explicit content from friends or contacts over social networks and asked them about how they typically react. Over two thirds of respondents had received unsolicited sexual content, and between 40% (females) and 60% (males) of respondents had received unsolicited violent content. There was no significant gender difference in likelihood of having received sexual content, but females were less likely to have received explicit violent content. Females rated both sexual and violent content, respectively, as significantly less funny, less exciting, and more disturbing than males did. This is consistent with research suggesting males show greater preferences for explicit media than females [11], suggesting that this effect extends to reactions to real-world footage of violent and sexual material exchanged within peer networks. It is worth noting that the current study used included a basic measure of gender, and the sample only included one non-binary participant, meaning that gender-related analyses focused simply on comparing those who self-identified as male with those who self-identified as female. Future research into this topic could benefit from including a gender identity scale (e.g., [16]) and recruiting a more diverse sample, in order to gain a more nuanced understanding of the role of gender identity and diversity in reactions to explicit sexual and violent content.

Regardless of gender, those who reported more frequent receipt of sexual content rated it as funnier than those who received it occasionally or never, and, similarly, those who reported previously receiving violent content rated it as funnier than those who had never received it. Females rated violent content as equally low in “excitement” value, regardless of whether they had been exposed to such content in the past, whereas males who were exposed many times rated such content as more exciting than those who had never received it, a result that was not specifically predicted, but may suggest a gender difference in changes in preference for explicit material over time.

Although participants with greater exposure rated some extreme content as more exciting and funnier than those with lower or no exposure, exposure did not influence how disturbing either violent or sexual content were rated; this contradicts what we might expect if a “desensitization” effect were at work. There are several possible explanations for this. Firstly, it is important to note that the phenomenon of desensitization has historically been recognized as being contentious [17], and recent studies have demonstrated no evidence of emotional blunting in people with extensive exposure to violent videogame content [18,19]. Secondly, it is possible that desensitization is not experienced in response to the type of content we enquired about in the current study, perhaps because of the nature of the content (graphic, real content), or because of the nature of exposure (unsolicited receipt). Thirdly, it may be that the extent of exposure within our sample was not sufficient to have induced desensitization, or that we did not have enough sensitivity in our measure of exposure (e.g., “multiple times” was the highest category, but this does not differentiate individuals who receive such content several times a day from those how have received it multiple times but over an extended period, and much less frequently); future research involving participants with a wide range of prior exposure to such content, with more detailed items to evaluate the nature and extent of exposure, would be valuable to address this limitation.

There are also multiple possible explanations for the differences in ratings of how funny and exciting explicit content were by those with different levels of exposure. Exposure may alter reactions over time (with people developing a “taste” for it, or learning to enjoy it), but, equally, people who feel more positively about such content may actively increase their exposure to it. Although we specifically asked about receipt of unsolicited content, someone who enjoys receiving explicit media might be more likely react positively, encouraging the sender and inducing further exposure. Furthermore, artificial intelligence algorithms within social media are likely to curate content for users based on the type of content that they view [20], which would further exacerbate arguably unsolicited exposure to such content, beyond that shared directly by friends or contacts. Alternatively, frequent recipients of unsolicited content may begin by disliking it, but adapt their attitude/reaction in response to cognitive dissonance, and/or to preserve relationships with the sender; one study found this type of reaction to unwanted physical gifts [21], and it is possible that similar psychological processes could occur in reaction to receiving unwanted digital content.

A cross-sectional piece of work such as the current study is unable to differentiate between these possible explanations, and further research of a qualitative or longitudinal nature would be valuable to learn more about this topic. There are a number of other limitations that future work could address; we used a convenience sample, approximately 75% of whom were female, and just over half of whom were students, so a more representative sample it would be beneficial to determine whether these effects are replicated in the wider population. We gave a fairly broad definition of explicit content, and participants may have varied considerably in the kind of content they pictured when rating their typical reactions. We did not seek to measure experiences of “sexting”—a complex behaviour that others have studied in depth (e.g., [22]); hence, the questions enquired about content from friends or contacts (rather than from romantic interests), but it is possible that some participants considered experiences of sexting when responding to the questions about sexual content. Thus, more specificity in the description of the behaviour being enquired about, and/or asking participants to give details about the type of content they receive, would be informative in future work. In addition, we focused solely on receipt of explicit content, but it is likely that there is, for some, a reciprocal exchange of such material, and future work into the sharing as well as the consumption of this type of content would be valuable.

This research was carried out prior to the recent murder of George Floyd in May 2020, and the widespread sharing of graphic footage of the tragic event over social and news media. The current study sought primarily to learn more about the phenomenon of casual sharing of explicit content amongst friends and contacts—in order to provoke a reaction, and/or under the guise of entertainment. However, recent events highlight the fact that, in other contexts, disturbing violent content can be shared for greatly differing reasons, for example, “virtually viewing police violence can also generate solidarity and… galvanize activism for police reform” [2] (p. 2); however, Boyd and Swanson also emphasize the potential for distress and discrimination associated with sharing content in such contexts, again highlighting the need for further research into the psychological consequences of unmonitored exposure to explicit violent content.

## 5. Conclusions

Despite a number of methodological limitations, the current study presents novel data on how people react to receiving unsolicited explicit content of a violent or sexual nature, from friends or contacts over social media networks, and highlights gender and prior exposure as factors that can play a significant role in these reactions. It also demonstrates that this kind of content is experienced as being “disturbing” by the majority of respondents: particularly violent content, which both males and females, and people with and without prior experience, rated as disturbing. Further research into the potentially harmful impact of exposure to such content, uncontrolled within the general population, is an important priority, because a better understanding of this area has the potential to assist in educating people about risks and in challenging perceived norms and in supporting individuals who may experience distress as a result of exposure to such content.

## Figures and Tables

**Table 1:** Social media platforms used by respondents.

Social Media Site	% of Sample Using Site
Facebook/Facebook messenger	94.1%
Whatsapp	81.4%
Instagram	62.1%
YouTube	58.4%
Google	50.9%
Snapchat	46.5%
Twitter	28.3%
LinkedIn	21.9%
Skype	11.2%
TikTok	10.4%
Other SM	3.7%

**Table 2:** Mean (SD) ratings of explicit content, by males and females.

Rating of:	Males Mean (SD)	Females Mean (SD)
Sexual content—funny	3.53 (1.80)	2.04 (2.02)
Sexual content—exciting	2.75 (1.85)	1.08 (1.43)
Sexual content—disturbing	3.32 (1.49)	4.56 (1.66)
Violent content—funny	1.55 (1.66)	0.71 (1.35)
Violent content—exciting	1.42 (1.62)	0.60 (1.13)
Violent content—disturbing	4.21 (1.83)	4.99 (1.72)

0 = strongly disagree; 1 = disagree; 2 = slightly disagree; 3 = neutral; 4 = slightly agree; 5 = agree; 6 = strongly agree.

## References

[1] Bender P.K., Plante C., Gentile D.A. (2018). The effects of violent media content on aggression. Curr. Opin. Psychol..

[2] Boyd R.W., Swanson W.S. (2016). The evolution of virtual violence: How mobile screens provide windows to real violence. Pediatrics.

[3] Thompson R.R., Jones N.M., Holman E.A., Silver R.C. (2019). Media exposure to mass violence events can fuel a cycle of distress. Sci. Adv..

[4] Brooks S. (2015). Does personal social media usage affect efficiency and well-being?. Comput. Hum. Behav..

[5] Wong D., Amon K.L., Keep M. (2019). Desire to Belong Affects Instagram Behavior and Perceived Social Support. Cyberpsychol. Behav. Soc. Netw..

[6] Weiss R., Samenow C.P. (2010). Smart phones, social networking, sexting and problematic sexual behaviors-a call for research. Sex. Addict. Compuls..

[7] Hayes R.M., Dragiewicz M. (2018). Unsolicited dick pics: Erotica, exhibitionism or entitlement?. Womens. Stud. Int. Forum.

[8] Paasonen S., Light B., Jarrett K. (2019). The Dick Pic: Harassment, Curation, and Desire. Soc. Media Soc..

[9] Oswald F., Lopes A., Skoda K., Hesse C.L., Pedersen C.L. (2019). I’ll Show You Mine so You’ll Show Me Yours: Motivations and Personality Variables in Photographic Exhibitionism. J. Sex Res..

[10] Wolak J., Mitchell K., Finkelhor D. (2007). Unwanted and wanted exposure to online pornography in a national sample of youth internet users. Pediatrics.

[11] Emmers-Sommer T.M., Pauley P., Hanzal A., Triplett L. (2006). Love, suspense, sex, and violence: Men’s and women’s film predilections, exposure to sexually violent media, and their relationship to rape myth acceptance. Sex Roles.

[12] Funk J.B. (2013). Media violence, desensitization, and psychological engagement. Oxford Handb. Media Psychol..

[13] Griffiths M.D., Shuckford G.L.J. (1989). Desensitization to television violence: A new model. New Ideas Psychol..

[14] Check J.V.P., Guloien T.H. (2012). Reported proclivity for coercive sex following repeated exposure to sexually violent pornography, nonviolent dehumanizing pornography, and erotica. Pornogr. Res. Adv. Policy Consid..

[15] Seto M.C., Maric A., Barbaree H.E. (2001). The role of pornography in the etiology of sexual aggression. Aggress. Violent Behav..

[16] Ho F., Mussap A.J. (2019). The Gender Identity Scale: Adapting the Gender Unicorn to measure gender identity. Psychol. Sex. Orientat. Gend. Divers..

[17] Funk J.B., Baldacci H.B., Pasold T., Baumgardner J. (2004). Violence exposure in real-life, video games, television, movies, and the internet: Is there desensitization?. J. Adolesc..

[18] Kühn S., Kugler D., Schmalen K., Weichenberger M., Witt C., Gallinat J. (2019). The Myth of Blunted Gamers: No Evidence for Desensitization in Empathy for Pain after a Violent Video Game Intervention in a Longitudinal fMRI Study on Non-Gamers. NeuroSignals.

[19] Szycik G.R., Mohammadi B., Hake M., Kneer J., Samii A., Münte T.F., Te Wildt B.T. (2017). Excessive users of violent video games do not show emotional desensitization: An fMRI study. Brain Imaging Behav..

[20] Spohr D. (2017). Fake news and ideological polarization: Filter bubbles and selective exposure on social media. Bus. Inf. Rev..

[21] Branco-Illodo I., Heath T., Tynan C. (2020). ‘You really shouldn’t have!’ Coping with failed gift experiences. Eur. J. Mark..

[22] Ringrose J., Gill R., Livingstone S., Harvey L. A Qualitative Study of Children, Young People and ‘Sexting’: A Report Prepared for the NSPCC. http://eprints.lse.ac.uk/44216/.

